# Correlations of Lumbar Interspinous Distance with Neuroforaminal Dimensions, Disc Space Height, and Patient Demographic Factors

**DOI:** 10.3390/tomography11090100

**Published:** 2025-08-27

**Authors:** Carson Cummings, Zachary Brandt, Kai Nguyen, Asael Isaac, Jean-Carlos Gutierrez, Ashley Kempf, David Cheng, Joel D. Carson, Emily Novak, Jacob Razzouk, Olumide Danisa, Wayne Cheng

**Affiliations:** 1 School of Medicine, Loma Linda University, Loma Linda, CA 92350, USA; ccummings@students.llu.edu (C.C.); zbrandt@students.llu.edu (Z.B.); kinguyen@students.llu.edu (K.N.); asisaac@students.llu.edu (A.I.); akempf@students.llu.edu (A.K.); enovak@students.llu.edu (E.N.); jrazzouk@students.llu.edu (J.R.); 2 Department of Orthopaedic Surgery, Loma Linda University Health, Loma Linda, CA 92354, USA; jeancarlosgu.14@gmail.com (J.-C.G.); davidwaynecheng@gmail.com (D.C.); 3 Department of Orthopaedic Surgery and Neurosurgery, Duke University Medical Center, Durham, NC 27710, USA; olumide.danisa@duke.edu; 4 Department of Orthopaedic Surgery, Jerry L. Pettis Veterans Affairs (VA) Medical Center, Loma Linda, CA 92357, USA

**Keywords:** lumbar spine, interspinous distance, neuroforamina, disc space, morphometric analysis, computed tomography

## Abstract

Background/Objectives: A thorough understanding of spinal anatomy is essential for diagnostic assessment and surgical intervention. Interspinous distance (ISD), neuroforaminal dimensions (NFDs), and disc space height (DSH) have each been studied separately; however, their interrelationship remains unstudied. Given the use of interspinous implants as a minimally invasive treatment for lumbar stenosis and degenerative disc disease, defining these relationships is of growing clinical significance. This study investigates the correlation between ISD and both NFDs and DSH in a normative population and whether ISD varies with demographic factors. Methods: A retrospective chart review was performed on 852 patients who underwent CT imaging of the lumbar spine. ISD was measured from L1 to L5 as the shortest distance between the most caudal tip of the superior spinous process and the inferior spinous process. DSH was measured at the anterior, middle, and posterior margins. NFDs were assessed in axial and sagittal views, including axial width, craniocaudal height, and foraminal area. Statistical analysis assessed correlations between ISD, NFDs, DSH, and demographic variables. Results: No strong correlation was observed between ISD and either NFDs or DSH. Slightly greater correlation was present at L1–L3, weakening at L4–L5, where interspinous implants are most commonly placed. Demographic analysis revealed no consistent relationship between ISD and ethnicity, sex, or BMI. While it may be expected that larger ISD correlates with greater NFDs or DSH, our findings do not support this assumption. Conclusions: ISD does not strongly correlate with NFDs or DSH, and demographic factors do not significantly influence ISD in a healthy population.

## 1. Introduction

Lumbar decompression, stabilization, and interspinous implant procedures rely on modifying spinal morphology to relieve symptoms associated with neural compression [1]. Therefore, a thorough understanding of spinal anatomy is essential for both diagnostic assessment and surgical intervention. While interspinous distance (ISD), neuroforaminal dimensions (NFDs), and disc space height (DSH) have each been studied separately [2,3,4,5], their relationship concerning size remains unstudied. Prior research has established normative values for NFDs [4] and investigated how these dimensions correlate to DSH and patient demographics [6]; however, there is limited data on how ISD correlates with these parameters. Given the use of interspinous implants as a minimally invasive treatment for lumbar stenosis and degenerative disc disease, defining these relationships is of growing clinical significance [7].

Interspinous implants expand the ISD to indirectly decompress neural structures, altering NFDs and DSH [8,9]. While studies have examined the effects of these implants [8,9], little is known about the baseline anatomical correlations between these structures. Additionally, demographic factors such as ethnicity, sex, and body mass index (BMI) may influence ISD, yet their impact remains undefined. Previous literature has demonstrated racial and anthropometric variability in other spinal parameters [4,5,6,10,11,12], but it remains unclear whether these factors affect ISD in a normative population.

This study investigates the correlation between ISD and both NFDs and DSH in a normative population. Additionally, we assess whether the ISD varies in relation to demographic factors. To our knowledge, this is the first study to evaluate these relationships in a large, healthy cohort. By defining these relationships, this research provides insights that may improve the precision of interspinous implant placement and improve patient outcomes in minimally invasive spinal surgery.

## 2. Materials and Methods

Following Institutional Review Board approval (#5250240), a retrospective chart review was conducted on patients who underwent lumbar spine computed tomography (CT) imaging at a single tertiary academic medical center between January 2013 and January 2023. All CT scans were acquired using a GE Discovery 750 HD 64-slice CT scanner. Patient consent was not required due to the retrospective and radiographic nature of the study. Patients were included if their CT imaging provided continuous visualization in both sagittal and axial views, extending from the superior endplate of L1 to the caudal tip of the L5 spinous process. Patients were excluded if they demonstrated any pathologic spinal anatomy, including congenital lumbar stenosis, spinal fracture, neuroforaminal stenosis, spinal malignancy, spinal infection, prior spinal surgery, congenital growth delays, or chromosomal abnormalities. After applying exclusion criteria and removing duplicate records, a final cohort of 852 patients was identified. All imaging data were reviewed using Enterprise Imaging version 8.3x (AGFA HealthCare, 2023, Mortsel, Belgium).

**Figure 1:**
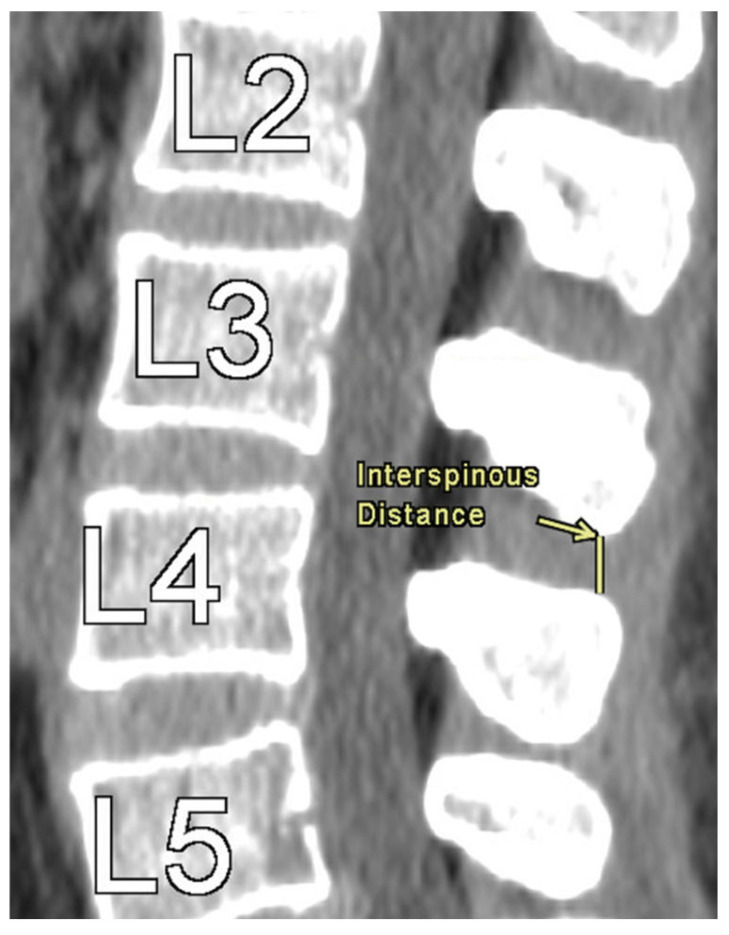
Interspinous Distance Measurement at L3–L4.

**Figure 2:**
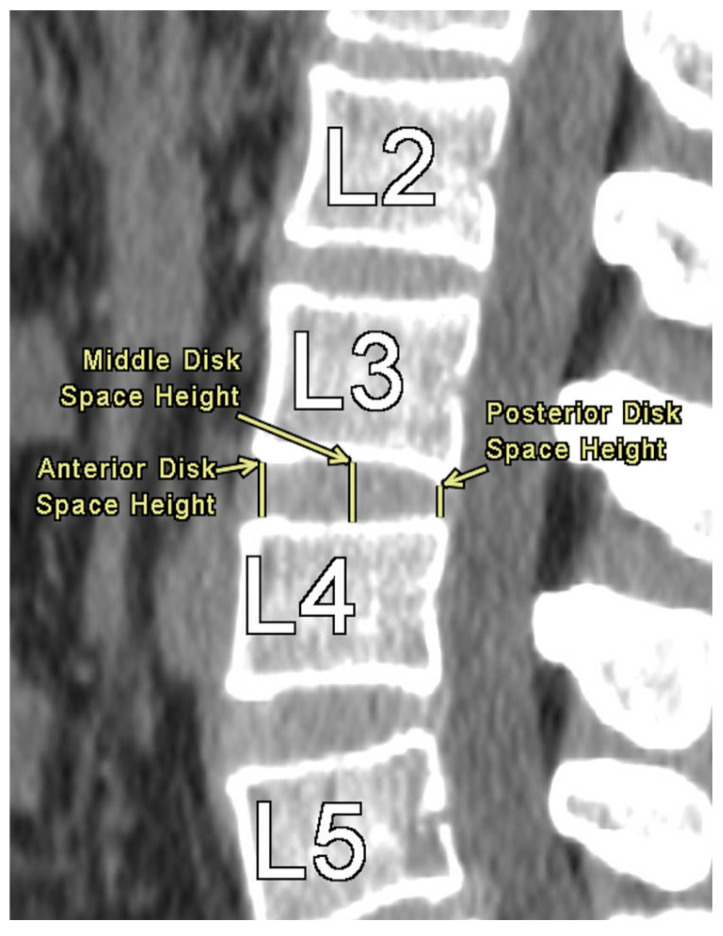
Disc Space Height Measurement at L3–L4.

The authors took axial and sagittal CT imaging measurements and first located the midline of the lumbar spine in both views. All measurements were performed by seven trained evaluators using a standardized protocol under the supervision of a board-certified musculoskeletal radiologist, with consistent anatomical landmarks used across evaluators to minimize measurement variability. Then, the measurements were taken at each level from L1 to L5. Figure 1 provides an illustration of the measurement technique for ISD, measured as the shortest vertically linear distance between the most caudal tip of the superior spinous process down to the top of the inferior spinous process in the mid-sagittal plane. DSH was measured between each vertebra using sagittal CT views and recorded in the mid-sagittal plane anteriorly, at the middle, and posteriorly. These distances were defined as the shortest vertical distances between the superior and inferior endplates at their respective vertebra, as illustrated in Figure 2.

**Figure 3:**
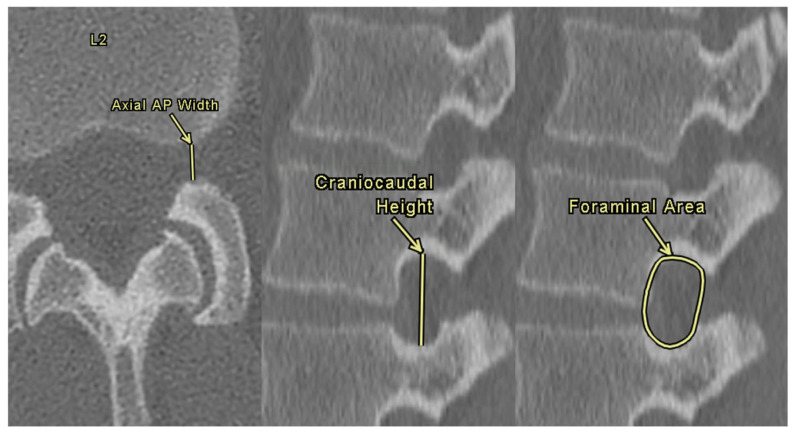
Neuroforaminal dimension measurements at L2 (**left** to **right**): axial AP width, craniocaudal height, and foraminal area.

NFDs were assessed bilaterally at each lumbar level using both axial and sagittal CT images. Axial anterior–posterior (AP) width was defined as the shortest distance between the posterior inferior corner of the superior vertebra and the anterior border of the superior articular process of the lower vertebra. Foraminal height was defined as the maximum vertical distance between the inferior border of the pedicle of the superior vertebra and the superior border of the pedicle of the inferior vertebra. The foraminal area was calculated by manually tracing the bony boundaries of the foramen on sagittal slices using the freeform area tool within the Enterprise Imaging 8.3.x platform (AGFA HealthCare, 2023, Mortsel, Belgium). These three measurements are demonstrated in Figure 3.

All statistical analyses were performed using R version 4.5.1 (R Core Team, 2025, Vienna, Austria). A two-sided significance threshold of *p* < 0.05 was applied for all tests. No imputation was performed for missing data; instead, missing values were handled using pairwise deletion to maximize the use of available data and preserve sample size, thereby maintaining statistical power. To assess associations between ISD and continuous morphometric variables, including DSH, NFDs, and BMI, Spearman’s rank correlation test was used due to the non-parametric distribution of the data. Spearman’s rank correlation coefficients ≥ ±0.7 were considered strong, those between ±0.4 and ±0.7 were considered moderate, those between ±0.1 and ±0.4 were considered weak, and values below ±0.1 were considered to indicate no correlation [13]. Sex-based differences in ISD were analyzed using the Mann–Whitney U test, a non-parametric alternative to the independent samples t-test. Differences across racial and ethnic groups were evaluated using the Kruskal–Wallis test. When statistically significant differences were observed, Dunn’s test was used for post hoc pairwise comparisons. For each comparison, the Hodges–Lehmann estimator was calculated to provide a non-parametric estimate of the median difference, which was reported along with 95% confidence intervals.

## 3. Results

After applying the exclusion criteria from the original imaging dataset, a total of 852 patients were included in the final study cohort. Included patients were between 18 and 38 years of age, with an average age of 27.87 ± 5.08 years. The cohort had a mean BMI of 28.21 ± 7.11, a mean height of 1.69 ± 0.11 m, and a mean weight of 80.46 ± 22.10 kg. The sex distribution was 54.3% female and 45.7% male. The population was racially and ethnically diverse, with 50.7% identifying as Hispanic/Latino, 33.6% as White, 11.4% as Black, and 4.4% as Asian.

Table A1 presents the means and standard deviations of ISDs measured at each lumbar level from L1 to L5. Table A2 summarizes the DSH measurements at the anterior, middle, and posterior locations of each level. Table A3 describes the bilateral NFDs, including axial AP width, craniocaudal height, and foraminal area, at each lumbar level. These tables are provided in Appendix A.

The correlation between ISD and both DSH and NFD measurements was assessed using Spearman’s rank correlation and is reported in Table 1 and Table 2, respectively. All correlation coefficients for ISD vs. DSH ranged from 0 to ±0.18, reflecting mostly no correlation with a few weak statistically significant correlations observed at the L2 middle DSH (ρ = −0.11, *p* < 0.01), the L3 and L4 posterior DSHs (ρ = 0.12, *p* < 0.01 for both), and the L5 anterior and middle DSHs (ρ = −0.18 and −0.13, *p* < 0.01, respectively). Remaining values showed no significant correlation (|ρ| < 0.1). In Table 2, nearly all ISD vs. NFD correlations were significant but very weak (ρ = −0.03 to 0.28), with a few values indicating no correlation. Most results were statistically significant, except for the L3 right AP width (*p* = 0.08), the L4 right AP width (*p* = 0.10), and both left and right L5 craniocaudal heights (*p* = 0.46 and 0.47, respectively). However, even where statistical significance was present, the strength of correlation remained uniformly weak, underscoring that significance does not imply clinical or anatomical relevance in this context.

Ethnic differences in ISD were evaluated using the Kruskal–Wallis test and are summarized in Table 3. Statistically significant variation was observed across ethnic groups at the L1 (*p* = 0.01) and L2 (*p* = 0.04) lumbar levels. No significant differences were found at L3, L4, or L5 (*p* > 0.05 for all). Post hoc pairwise comparisons using Dunn’s test with Hodges–Lehmann estimates are presented in Table 4. At L1, ISD was significantly smaller in Black patients compared to White patients (Hodges–Lehmann estimate: −1.80, 95% CI: −2.90 to −0.70, *p* = 0.01). Similarly, at L2, Black patients demonstrated significantly smaller ISD values than White patients (HL estimate: −1.40, 95% CI: −2.40 to −0.30, *p* = 0.048). All other pairwise comparisons at both levels were not statistically significant after adjustment for multiple comparisons (*p* > 0.05).

Sex-based comparisons of ISD were assessed using the Mann–Whitney U test and are summarized in Table 5. Statistically significant differences were observed between male and female patients at the L1 (*p* = 0.04) and L5 (*p* < 0.01) lumbar levels. At L1, males had higher ISD values (median: 12.30 mm) compared to females (median: 11.60 mm), with a Hodges–Lehmann estimate of −0.60 mm (95% CI: −1.20 to 0.00). At L5, males again showed higher ISD values (median: 6.00 mm vs. 5.40 mm), with a Hodges–Lehmann estimate of −0.70 mm (95% CI: −1.10 to −0.30). No significant differences were observed at L2, L3, or L4 (*p* > 0.05 for all).

The relationship between BMI and ISD was evaluated using Spearman’s Rank correlation and is presented in Table 6. A weak negative correlation was observed at L1 (ρ = −0.09, *p* = 0.05), indicating a slight trend toward lower ISD values with higher BMI. No significant correlations were found at L2 through L5 (*p* ≥ 0.14 for all), and all correlation coefficients were below ±0.1, indicating no meaningful association between BMI and ISD at these levels.

## 4. Discussion

This study is the first to systematically investigate the relationship between ISD, NFDs, and DSH across the lumbar spine in a large, healthy cohort. Prior studies have established normative values for these lumbar morphometric features independently [2,3,4,5], but none have directly assessed how ISD correlates with surrounding anatomical structures. Given the increasing use of interspinous implants to treat lumbar stenosis and disc degeneration [14,15], understanding the anatomical context of ISD is of growing clinical importance.

No strong correlation was observed between ISD and either NFD or DSH measurements. Although it may seem intuitive that a larger ISD would correspond to greater foraminal or disc space dimensions, as has been shown in other regions of the spine [16,17], our findings do not support this assumption. Across all five lumbar levels, correlation coefficients were consistently weak or absent, with only a handful reaching statistical significance. These results suggest that ISD alone may not be a reliable marker for predicting adjacent morphometric dimensions under normal anatomical conditions. Although several relationships were statistically significant, the strength of correlation remained uniformly weak, reinforcing the importance of distinguishing statistical from anatomical or clinical relevance.

Interestingly, the few weak correlations that did emerge were more commonly observed at upper lumbar levels, L1 and L3. In contrast, levels L4 and L5, the most frequent targets for interspinous implant placement, showed minimal to no correlation [8]. This finding may reflect both anatomical and technical factors. Spinal curvature and increased lordosis at the lower lumbar spine can distort sagittal imaging planes, potentially introducing measurement errors [18]. Alternatively, the reduced correlation at these levels may represent a genuine anatomical dissociation between interspinous and adjacent structures, underscoring the importance of careful, level-specific evaluation when planning surgical interventions.

Demographic analysis further underscored the variability and complexity of ISD. Unlike previous studies that demonstrated racial and anthropometric variability in spinal morphology [4,5,6,10,11,12], our findings suggest that ISD remains relatively stable across populations under normal anatomic conditions. Sex-based comparisons demonstrated statistically larger ISDs in males at L1 and L5; however, differences were minor in magnitude and not observed consistently across all levels, reinforcing that ISDs do not significantly vary between males and females [10]. Although significant differences in ISD were noted between Black and White patients at L1 and L2, no clear ethnic trends were identified overall [19]. Lastly, no meaningful correlation was found between BMI and ISD at any level, differing from prior studies linking higher BMI to morphometric variation in the lumbar spine [20]. These findings indicate that the ISD remains relatively stable across sex, racial, and anthropometric groups in healthy individuals.

Our findings challenge the intuitive assumption that greater ISD corresponds to larger NFDs or DSHs. This concept is particularly relevant for surgical practices that rely on interspinous implants designed to indirectly decompress neural elements by increasing ISD [14]. Given our findings that ISD does not strongly correlate with NFD or DSH in asymptomatic patients, device sizing and positioning should not rely solely on ISD. Moreover, the weak and inconsistent associations at the most clinically utilized levels, L4 and L5, underscore the need for specific radiographic assessment of target structures prior to surgical planning [8]. Given the normative population studied, it remains unclear whether similar patterns would be observed in the presence of pathology, such as lumbar stenosis, spondylolisthesis, disc degeneration, or synovial cysts, where anatomical relationships may shift [21,22,23]. Additionally, future studies should also assess how these relationships change following surgical intervention, as postoperative alterations in ISD may influence adjacent morphometric parameters.

This study has several limitations that should be taken into account when interpreting the results. As a retrospective analysis conducted at a single tertiary care center, the findings may not be generalizable to broader populations. While this study aimed to define normative anatomical relationships, our dataset may reflect demographic or institutional biases not present elsewhere. The analysis was limited to patients aged 18 to 38 years with normal lumbar anatomy to isolate baseline relationships between ISD, NFDs, and DSH. Although this approach minimizes confounding from degenerative pathology, it also restricts the applicability of the study’s findings to clinical populations in which spinal disease is more prevalent [24]. Additionally, anatomical distortion from lumbar lordosis and the sagittal curvature of the lower spine may have introduced variability in measurement accuracy, particularly at the L4 and L5 levels. These levels are especially relevant to interspinous implant placement but are also more susceptible to error due to imaging angle and alignment [18]. Although all measurements were performed using a standardized protocol under the supervision of a board-certified musculoskeletal radiologist, subtle variability between measurement takers may still have influenced the results.

## 5. Conclusions

This study is the first to systematically examine the relationship between ISD, NFDs, and DSH across the lumbar spine in a healthy adult population. Contrary to common assumptions, ISD did not strongly correlate with either NFDs or DSH at any level, suggesting it may not be a reliable proxy for these spinal parameters under normal anatomical conditions. Weak correlations observed at upper lumbar levels were not present at L4 and L5, the levels most commonly targeted by interspinous implants [8]. Demographic variables, including sex, ethnicity, and BMI, showed minimal and inconsistent associations with ISD. These findings underscore the importance of individualized assessment, rather than relying solely on ISD, in clinical decision-making and device planning. Future research should investigate whether these relationships change in the presence of pathology or postoperatively to better inform surgical strategies and implant design.

## Figures and Tables

**Table 1:** Spearman’s rank correlation between interspinous distance and disc space height measurements.

Level	Measurements	ρ (Spearman’s Correlation)	95% Confidence Interval		*p*-Value
			Lower Bound	Upper Bound	
L1	Anterior Disc Space Height	0.02	−0.06	0.11	0.56
	Middle Disc Space Height	−0.03	−0.11	0.05	0.46
	Posterior Disc Space Height	0.07	0.00	0.15	0.07
L2	Anterior Disc Space Height	−0.05	−0.12	0.04	0.26
	Middle Disc Space Height	−0.11	−0.19	−0.02	<0.01
	Posterior Disc Space Height	0.07	−0.01	0.15	0.09
L3	Anterior Disc Space Height	0.00	−0.08	0.08	0.97
	Middle Disc Space Height	−0.01	−0.09	0.07	0.85
	Posterior Disc Space Height	0.12	0.04	0.20	<0.01
L4	Anterior Disc Space Height	−0.01	−0.09	0.07	0.77
	Middle Disc Space Height	0.01	−0.07	0.09	0.78
	Posterior Disc Space Height	0.12	0.04	0.20	<0.01
L5	Anterior Disc Space Height	−0.18	−0.25	−0.10	<0.01
	Middle Disc Space Height	−0.13	−0.21	−0.04	<0.01
	Posterior Disc Space Height	−0.05	−0.13	0.04	0.26

**Table 2:** Spearman’s rank correlation between interspinous distance and neuroforaminal measurements.

Level	Measurements	ρ (Spearman’s Correlation)	95% Confidence Interval		*p*-Value
			Lower Bound	Upper Bound	
L1 Left	Axial AP Width	0.13	0.05	0.21	<0.01
	Craniocaudal Height	0.17	0.08	0.26	<0.01
	Foraminal Area	0.26	0.18	0.33	<0.01
L1 Right	Axial AP Width	0.12	0.04	0.20	<0.01
	Craniocaudal Height	0.17	0.09	0.25	<0.01
	Foraminal Area	0.28	0.20	0.35	<0.01
L2 Left	Axial AP Width	0.16	0.08	0.24	<0.01
	Craniocaudal Height	0.14	0.06	0.22	<0.01
	Foraminal Area	0.19	0.10	0.27	<0.01
L2 Right	Axial AP Width	0.16	0.08	0.24	<0.01
	Craniocaudal Height	0.14	0.06	0.23	<0.01
	Foraminal Area	0.20	0.12	0.28	<0.01
L3 Left	Axial AP Width	0.09	0.01	0.17	0.03
	Craniocaudal Height	0.11	0.03	0.20	<0.01
	Foraminal Area	0.18	0.09	0.26	<0.01
L3 Right	Axial AP Width	0.07	0.00	0.16	0.08
	Craniocaudal Height	0.12	0.03	0.19	<0.01
	Foraminal Area	0.16	0.08	0.24	<0.01
L4 Left	Axial AP Width	0.08	0.00	0.16	0.05
	Craniocaudal Height	0.11	0.03	0.19	<0.01
	Foraminal Area	0.13	0.05	0.21	<0.01
L4 Right	Axial AP Width	0.07	−0.01	0.15	0.10
	Craniocaudal Height	0.12	0.04	0.20	<0.01
	Foraminal Area	0.16	0.08	0.24	<0.01
L5 Left	Axial AP Width	0.12	0.03	0.20	<0.01
	Craniocaudal Height	0.03	−0.05	0.11	0.46
	Foraminal Area	0.14	0.06	0.22	<0.01
L5 Right	Axial AP Width	0.11	0.03	0.19	<0.01
	Craniocaudal Height	−0.03	−0.11	0.05	0.47
	Foraminal Area	0.17	0.09	0.25	<0.01

**Table 3:** Kruskal–Wallis test comparing interspinous distance measurements across ethnic groups at each lumbar level.

Level	Caucasian		Hispanic/Latino		African American		Asian		*p*-Value
	Median	IQR	Median	IQR	Median	IQR	Median	IQR	
L1	12.60	5.08	11.70	4.60	10.70	5.08	12.45	2.83	0.01
L2	10.50	4.40	10.30	4.00	9.45	5.58	11.00	6.00	0.04
L3	7.20	4.30	7.50	3.65	6.65	4.68	7.75	3.48	0.18
L4	5.70	3.40	5.80	3.20	4.95	3.85	6.00	3.50	0.16
L5	5.80	3.30	5.80	3.20	5.05	3.43	6.20	4.10	0.41

**Table 4:** Dunn’s post hoc pairwise comparisons of ISD measurements by ethnicity with Hodges–Lehmann estimates and 95% confidence intervals.

Comparison Groups	Level	Hodges–Lehmann Estimate	95% Confidence Interval		Adjusted *p*-Value
			Lower Bound	Upper Bound	
Asian VS Black	L1	1.10	−0.50	2.80	1.00
	L2	1.90	0.10	3.60	0.17
Asian VS Hispanic/Latino	L1	0.30	−1.00	1.50	1.00
	L2	0.70	−0.70	2.20	1.00
Asian VS White	L1	−0.50	−2.00	0.80	1.00
	L2	0.50	−1.00	2.00	1.00
Black VS Hispanic/Latino	L1	−0.90	−1.90	0.10	0.54
	L2	−1.10	−2.10	−0.10	0.18
Black VS White	L1	−1.80	−2.90	−0.70	0.01
	L2	−1.40	−2.40	−0.30	0.05
Hispanic/Latino VS White	L1	−0.80	−1.50	−0.20	0.06
	L2	−0.30	−0.80	0.30	1.00

**Table 5:** Mann–Whitney U test results comparing interspinous distance between male and female patients at each lumbar level, including Hodges–Lehmann estimates and confidence intervals.

Level	Female		Male		Hodges–Lehmann Estimator	95% Confidence Interval		*p*-Value
	Median	IQR	Median	IQR		Lower Bound	Upper Bound	
L1	11.6	4.8	12.3	4.45	−0.60	−1.20	−0.00003	0.04
L2	10.2	4.2	10.5	4.65	−0.40	−0.90	0.20	0.16
L3	7.5	4.1	7.2	3.7	0.10	−0.40	0.60	0.70
L4	5.65	3.6	5.8	3.4	−0.30	−0.70	0.10	0.17
L5	5.4	3.1	6	3.6	−0.70	−1.10	−0.30	<0.01

**Table 6:** Spearman’s rank correlation coefficients assessing the relationship between BMI and ISD at each lumbar level, with corresponding confidence intervals.

Level	ρ (Spearman’s Correlation)	95% Confidence Interval		*p*-Value
		Lower Bound	Upper Bound	
L1	−0.09	−0.17	−0.003	0.051
L2	−0.07	−0.15	0.02	0.14
L3	−0.001	−0.09	0.09	0.98
L4	0.07	−0.02	0.14	0.14
L5	−0.00002	−0.08	0.09	1.00

## Data Availability

The original contributions presented in this study are included in the article. Further inquiries can be directed to the corresponding author(s).

## Abbreviations

The following abbreviations are used in this manuscript:

ISD	Interspinous Distance
DSH	Disc Space Height
NFD	Neuroforaminal Dimension
AP	Anterior–Posterior
BMI	Body Mass Index
CT	Computed Tomography

## Appendix A

**Table A1:** Interspinous Distance Descriptive Values.

Level	Mean (mm)	SD
L1	11.91	3.47
L2	10.29	3.14
L3	7.76	2.95
L4	6.08	2.55
L5	6.21	2.8

**Table A2:** Disc Space Height Descriptive Values.

Level	Variable	Mean (mm)	SD
L1	Anterior DSH	5.54	1.75
	Middle DSH	6.63	1.35
	Posterior DSH	3.15	1.19
L2	Anterior DSH	6.46	1.81
	Middle DSH	8.02	1.58
	Posterior DSH	4.13	1.27
L3	Anterior DSH	7.71	1.86
	Middle DSH	8.94	1.57
	Posterior DSH	4.73	1.40
L4	Anterior DSH	9.19	2.06
	Middle DSH	9.43	1.69
	Posterior DSH	4.91	1.53
L5	Anterior DSH	10.68	2.71
	Middle DSH	8.24	1.97
	Posterior DSH	3.80	1.45

**Table A3:** Neuroforaminal Dimensions Descriptive Values.

Level/Side	Variable	Mean (mm)	SD
L1 Left	Axial AP Width	9.20	2.07
	Craniocaudal Height	17.53	2.23
	Foraminal Area	129.71	31.37
L1 Right	Axial AP Width	9.19	2.04
	Craniocaudal Height	17.46	2.22
	Foraminal Area	130.63	31.45
L2 Left	Axial AP Width	8.93	1.90
	Craniocaudal Height	18.63	2.20
	Foraminal Area	139.79	33.98
L2 Right	Axial AP Width	9.04	1.97
	Craniocaudal Height	18.64	2.33
	Foraminal Area	141.38	33.85
L3 Left	Axial AP Width	8.74	1.92
	Craniocaudal Height	18.73	2.39
	Foraminal Area	140.13	33.97
L3 Right	Axial AP Width	8.73	1.87
	Craniocaudal Height	18.54	2.38
	Foraminal Area	139.46	34.20
L4 Left	Axial AP Width	8.09	1.89
	Craniocaudal Height	18.06	2.55
	Foraminal Area	131.79	32.20
L4 Right	Axial AP Width	8.10	2.00
	Craniocaudal Height	17.80	2.37
	Foraminal Area	130.44	30.88
L5 Left	Axial AP Width	8.23	2.33
	Craniocaudal Height	15.63	2.65
	Foraminal Area	118.04	31.09
L5 Right	Axial AP Width	8.51	2.37
	Craniocaudal Height	15.55	2.63
	Foraminal Area	119.85	30.92
